# Subwavelength hybrid plasmonic structures for nonlinear nanophotonics

**DOI:** 10.1038/s41377-021-00479-9

**Published:** 2021-02-22

**Authors:** Ann-Katrin U. Michel

**Affiliations:** Optical Materials Engineering Laboratory, Department of Mechanical and Process Engineering, ETH Zurich, Zurich, Switzerland

**Keywords:** Microresonators, High-harmonic generation

## Abstract

Plasmonic structures made of a semiconductor-insulator-metal hybrid provide efficient routes for second-harmonic and sum-frequency generation in sub-micrometer structures, which ultimately may boost on-chip integrated plasmonic circuits.

Approximately 50 years ago, the evolution of integrated photonics was predicted to be quite similar to Moore’s law in electronics, although featuring a different slope.^1^ Since then, photonic integrated circuits have become an established technology for transporting and processing information. In these circuits, linear and nonlinear operations are executed by different generic optical components, such as optical amplifiers, phase shifters, polarization converters, and waveguides.^2^ Recent breakthroughs in programmable photonics for deep learning and quantum applications^3,4^ give a glimpse of the potential for light control that such a platform can provide.

However, to bring off-the-shelf programmable photonics within reach, several technological challenges need to be overcome. Size reduction and increasing integration complexity, as well as thermal management, are among the major issues that require smart solutions. Design innovations and technological developments are necessary not only to realize large wafer-scale fabrication but also to integrate complex or potentially new functionalities into state-of-the-art indium phosphide, silicon(-on-insulator), and silicon nitride platforms.^2,5–7^

One of the cornerstones for achieving functionalities such as optical-frequency synthesis or higher-order-mediated frequency combs is second-order nonlinear optics.^7^ On-chip integration of nonlinear optics enables high-performance and compact platforms for frequency conversion for a variety of applications, such as optical communication, sensing, signal processing, and biophotonics. In particular, deep learning and quantum information processing require functional elements with a nonlinear response.^3^

Sum-frequency generation (SFG) is a second-order nonlinear optical process during which two photons annihilate and generate a single photon at a different frequency. In the special case of second-harmonic generation (SHG), the annihilation of two photons of the same frequency generates a photon with twice the energy. Hybrid plasmonic waveguides are a promising platform for SHG with high conversion efficiency.^8^ To this end, not only highly efficient optical interactions but also small footprints are essential for compatibility between nonlinear optics and nanophotonic architectures in integrated optics. While microstructures based on nonlinear materials have led to demonstrations of high conversion efficiencies, metallic nanostructures allow for miniaturization due to their tiny mode volumes. However, to achieve the “best of both worlds” for nonlinear optical interactions in hybrid plasmonic systems, the intrinsically high propagation loss and short coherence length in metals must be overcome. Then, the momentum conservation and phase-matching criteria necessary to ensure the constructive superposition of the generated higher harmonics can be met.^9^

Now, writing in this issue of *Light: Science & Applications*, Zhe Li and colleagues at the University of Limerick in Ireland, Wuhan University in China, and EPFL in Switzerland integrate a waveguide made of the nonlinear semiconductor aluminum gallium indium phosphide (AlGaInP) with a resonant plasmonic cavity based on a thin alumina and silver film.^10^ The lithographically defined AlGaInP layer is only ~110 nm thick, ~8% of the fundamental wavelength at 1.3 μm. The authors have already demonstrated low-threshold lasing in the red spectral range with a similar hybrid plasmonic system.^11^

In the work presented here, the authors adapt the system for efficient SHG and SFG. They excite highly confined transverse magnetic (TM) hybrid plasmonic modes at telecommunication wavelengths in the subwavelength waveguide. The needed TM hybrid mode can be excited due to symmetry breaking at the waveguide ends, thereby satisfying momentum conservation. Further miniaturization is studied by replacing the waveguide with a 1 μm disk. Its subwavelength dimension is needed for excitation of the TM modes.

By comparison with the hybrid system, a corresponding purely dielectric system in which a glass substrate replaces the plasmonic cavity shows almost undetectable SHG and SFG. Therefore, the much stronger field confinement and electric field enhancement of plasmonic systems are confirmed to be essential for high nonlinear conversion efficiencies.

With the presented work, Zhe Li and coauthors demonstrate a successful approach for the fabrication of submicron plasmonic hybrid waveguides and disks, both of which permit very efficient infrared-to-visible conversion (Fig. 1). Mode areas and volumes reduced to λ^2^/135 and λ^3^/206, as well as record-high SHG and SFG conversion efficiencies, have been achieved for fundamental wavelengths between 1.3 and 1.6 μm. While indications of the potential of hybrid plasmonic systems have been seen in previous studies,^8,11^ the present work not only confirms these prospects but also extends the principle to the telecom wavelength range. Furthermore, the established top-down lithography process applied by Zhe Li et al. seems crucial for future on-chip integration. Hence, the proposed platform could be a significant contribution to the development of a fundamental component of integrated nonlinear optics. Nevertheless, further optimization of the hybrid system, in regard to field enhancement and mode confinement, for example, will be needed. In the future, the implementation of broadband and widely tunable approaches^12^ could supplement or even replace wavelength-specific resonant mechanisms such as the presented hybrid plasmonic concept.

**Fig. 1:**
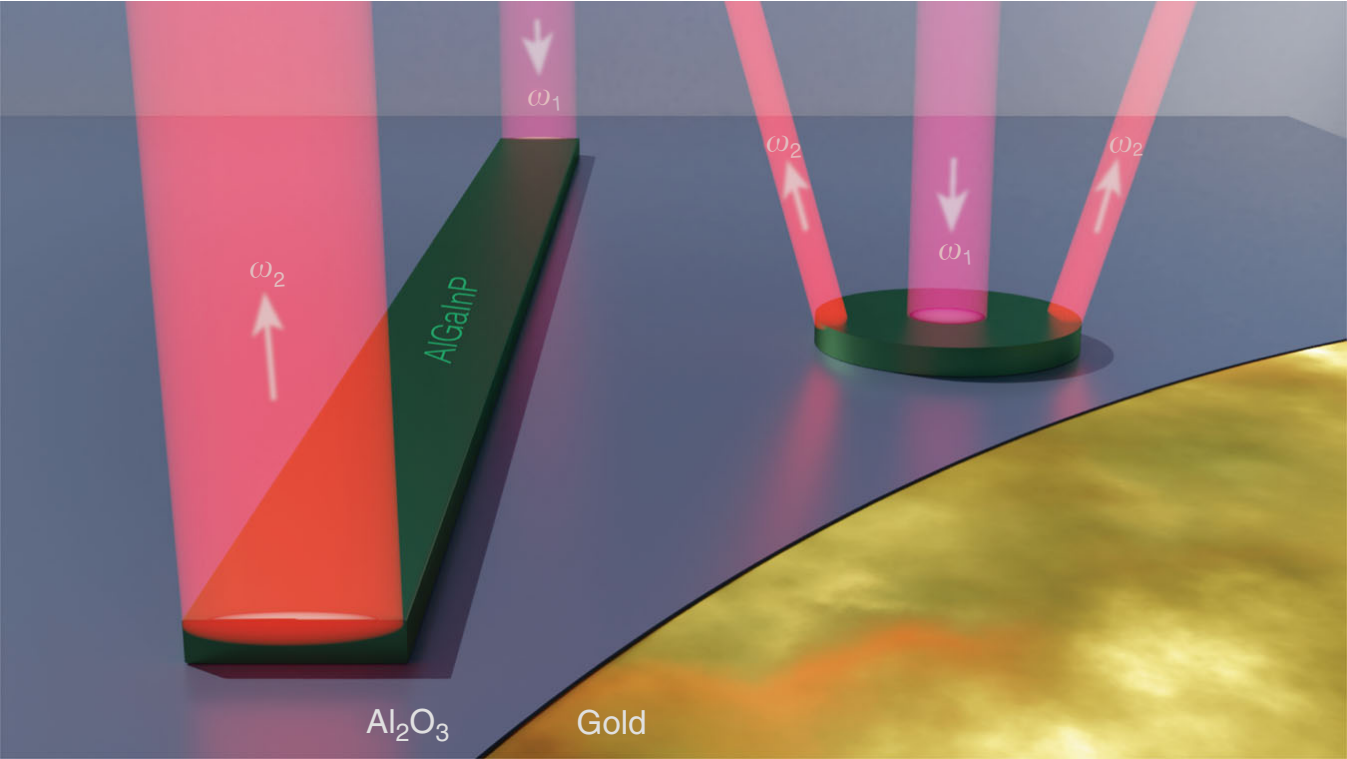
Schematic view of the hybrid plasmonic nanostructures for second-harmonic and sum-frequency generation.
